# Increased activity and expression of histone deacetylase 1 in relation to tumor necrosis factor-alpha in synovial tissue of rheumatoid arthritis

**DOI:** 10.1186/ar3071

**Published:** 2010-07-07

**Authors:** Tomoko Kawabata, Keiichiro Nishida, Koji Takasugi, Hiroko Ogawa, Kenei Sada, Yasutaka Kadota, Junko Inagaki, Satoshi Hirohata, Yoshifumi Ninomiya, Hirofumi Makino

**Affiliations:** 1 Department of Medicine and Clinical Science, Okayama University Graduate School of Medicine, Dentistry and Pharmaceutical Sciences, 2-5-1 Shikata-cho, Okayama City, Okayama 700-8558, Japan; 2 Department of Human Morphology, Okayama University Graduate School of Medicine, Dentistry and Pharmaceutical Sciences, 2-5-1 Shikata-cho, Okayama City, Okayama 700-8558, Japan; 3 Department of Medicine, Kurashiki Kosai Hospital, 5-4-16 Higashiduka, Kurashiki Okayama, 712-8044, Japan; 4 Department of Molecular Biology and Biochemistry, Okayama University Graduate School of Medicine, Dentistry and Pharmaceutical Sciences, 2-5-1 Shikata-cho, Okayama City, Okayama 700-8558, Japan; 5 Department of Orthopaedic Surgery, Okayama University Graduate School of Medicine, Dentistry and Pharmaceutical Sciences, 2-5-1 Shikata-cho, Okayama City, Okayama 700-8558, Japan

## Abstract

**Introduction:**

The purpose of this study was to investigate the profile of histone deacetylase (HDAC) expression in the synovial tissue of rheumatoid arthritis (RA) compared with that of normal control and osteoarthritis (OA), and to examine whether there is a link between HDAC activity and synovial inflammation.

**Methods:**

HDAC activity and histone acetyltransferase (HAT) activity were determined in nuclear extracts of total synovial tissue surgically obtained from normal, OA and RA joints. The level of cytoplasmic tumor necrosis factor a (TNFα) fraction was measured by ELISA. Total RNA of synovial tissue was used for RT-PCR of HDAC1-8. In synovial fibroblasts from RA (RASFs), the effects of TNFα on nuclear HDAC activity and class I HDACs (1, 2, 3, 8) mRNA expressions were examined by quantitative real-time PCR. The protein expression and distribution of class I HDACs were examined by Western blotting.

**Results:**

Nuclear HDAC activity was significantly higher in RA than in OA and normal controls and correlated with the amount of cytoplasmic TNFα. The mRNA expression of HDAC1 in RA synovial tissue was higher than in OA and normal controls, and showed positive correlation with TNFα mRNA expression. The protein level of nuclear HDAC1 was higher in RA synovial tissue compared with OA synovial tissue. Stimulation with TNFα significantly increased the nuclear HDAC activity and HDAC1 mRNA expression at 24 hours and HDAC1 protein expression at 48 hours in RASFs.

**Conclusions:**

Our results showed nuclear HDAC activity and expression of HDAC1 were significantly higher in RA than in OA synovial tissues, and they were upregulated by TNFα stimulation in RASFs. These data might provide important clues for the development of specific small molecule HDAC inhibitors.

## Introduction

Rheumatoid arthritis (RA) is an autoimmune disease characterized by chronic inflammation of the synovial tissues in multiple joints that leads to bone and joint destruction. Recent clinical application of biologic agents targeted to inflammatory cytokines including tumor necrosis factor α (TNFα) or interleukin-1β (IL)-1β dramatically changed the treatment strategy for RA. These molecular therapies of RA are more effective than the conventional disease-modifying anti-rheumatic drugs (DMARDs), and can even stop the destructive process in some RA patients [1]. Nevertheless, the etiology of RA inflammation still remains unknown, and there is a demand for developing new therapies with alternative targets.

The characteristic pathology of the RA synovial membrane, including synovial cell proliferation, and persistent recruitment, activation, retention and survival of infiltrated immune cells, might require epigenetic regulation of gene transcription, such as acetylation, methylation and ubiquitination [2]. Among these, histone modification through reversible acetylation is a crucial event in gene expression [3]. Histone acetylation is controlled by two enzymes, histone acetyltransferase (HAT) and histone deacetylase (HDAC) [4,5]. Mammalian HDACs are classified into two major classes [6]. Class I HDACs (HDACs 1, 2, 3, 8) are homologues of yeast PRD3 and are found exclusively in the nucleus. Class II HDACs (HDACs 4 to 7 and 9), homologues of yeast Hda1, are found in both the nucleus and the cytoplasm. Gene regulation by HDAC/HAT is complex, because the inhibition of HDAC activity results both in induction and repression of gene expression, depending on the cell types and cell lines [7-11]. Recent studies on the balance of HAT and HDAC activity in human RA synovial tissue indicated that HDAC activity was significantly decreased in RA synovial tissue compared to osteoarthritis (OA) and normal tissues, thus HDAC/HAT might be strongly shifted toward histone hyperacetylation in RA patients [12].

Inhibitors of HDACs, originally developed as anti-cancer agents, exhibit anti-proliferative activity of the cells through multiple mechanisms, such as induction of apoptosis, cell cycle arrest, and promotion of cell differentiation, via modulation of gene expression [13]. It was reported that HDAC inhibitors can also reduce the expression of inflammatory mediators, such as TNFα, IL-1β, IL-6, IL-8, transforming growth factor-β, and nitric oxide that are involved in the pathogenesis of inflammatory diseases [13-18]. We have reported recently that FK228, an inhibitor of class I HDAC shows inhibitory effects on the proliferation of synovial fibroblasts from RA (RASFs) and ameliorates collagen antibody-induced pathology in mice [19]. The inhibition of cell proliferation by FK228 treatment was accompanied by the induction of p16^INK4a ^and the up-regulation of p21^WAF1/Cip1 ^expression in RASFs. Moreover, the expression of TNFα and IL-1β was markedly reduced in the synovium of mice treated by FK228. However, it remains unknown which HDACs are specifically involved in the process of RA inflammation. This information would be necessary for the development of new drugs that would avoid adverse side-effects including haematological toxicity and gastrointestinal symptoms [20,21]. It is unclear why the inhibition of HDAC ameliorates experimentally-induced arthritis [19,22] if HDAC/HAT is shifted toward histone hyperacetylation [12].

Here we investigated the expression profiles of class I and II HDACs (HDAC 1-8) in OA and RA synovial tissues, to identify the candidate HDAC gene in synovial inflammation in RA. We examined HAT and HDAC activities in the total nuclear extracts of synovial tissues from RA patients predominantly treated with conventional DMARDs, and their relationship with the cytoplasmic level of TNFα. Our data might provide new leads toward future developments of specific HDAC inhibitors for epigenetic regulation of RA.

## Materials and methods

### Patients and tissue sampling

We obtained total synovial tissue specimens from 15 RA and 13 OA patients, and 3 normal control patients undergoing orthopedic surgery at Okayama University Hospital, with informed consent from the patients. All RA patients fulfilled the 1987 revised criteria of the American Rheumatism Association [23]. The study protocol is approved by Okayama University Institutional Review Board (accredited ISO9001/2000) and by local ethic committees at respective institute where available. Normal synovial tissues were obtained from amputation surgery for a malignant tumor (n = 1), and ligament reconstruction surgery (n = 2). Baseline characteristics of the patients are summarized in Table 1. Most RA patients were receiving nonsteroidal anti-inflammatory drugs (NSAIDs), oral corticosteroid at ≤7.5 mg, and DMARDs such as methotrexate (MTX) and sulfasalazine, but not anti-TNFα treatment. RA patients had active disease, as indicated by increased acute-phase reactants (serum C-reactive protein (CRP) level 14.3 ± 4.1 mg/liter). OA patients and normal controls exhibited no evidence of inflammatory response. Fresh synovial tissues were divided immediately into approximate portions of 333 mg for measurement of HDAC activity, and 30 mg for real-time RT-PCR analysis and stored at -80°C.

**Table 1:** Characteristics of the study subjects

	Normal	OA	RA
Age mean (yr)	19.3 ± 5.0	75.9 ± 1.0	65.4 ± 2.2
No. of patients	3	13	15
Sex (F/M)	0/3	13/0	14/1
Disease duration (yr)	N/A	8.6 ± 2.2	15.9 ± 2.7
CRP (mg/dl)	0.2 ± 0.1	0.1 ± 0.4	1.4 ± 0.4
Medication (no of patients)			
Oral corticosteroid (mg/dl)	0	0	11(4.3 ± 0.5)
Methotrexate (mg/day)	0	0	9 (6.8 ± 0.8)
DMARDs others	0	0	6
NSAIDs	0	5	9

OA, osteoarthritis; RA, rheumatoid arthritis; NA, not applicable; Values are means ± SE.

### Isolation and culture of human RASFs

Fresh synovial tissues were obtained, with written permission from three RA patients during total joint replacement surgery. The tissues were minced and then immediately digested with 0.2% collagenase (Wako, Osaka, Japan) and DNAase (Sigma-Aldrich St. Louis, MO, USA) at 37°C, as previously described [24]. Tissue debris was removed with a cell strainer, and the remaining cells were washed twice with medium consisting of Dulbecco's modified Eagle's medium (DMEM; Sigma-Aldrich) supplemented with 10% HEPES (Life Technologies, Tokyo, Japan), 100 IU penicillin/ml, and 100 mg of streptomycin/ml (Life Technologies). The resultant single cells were dispensed into the wells of a 24-well microtiter plate (Costar, Cambridge, MA, USA) at a density of 2 × 10^6 ^cells/ml in 2 ml of DMEM supplemented with 10% fetal bovine serum (FBS), 100 IU of penicillin/ml, and 100 mg of streptomycin/ml. The plates were incubated at 37°C in a humidified atmosphere containing 5% CO_2_. Synovial cell cultures were divided once weekly until the primary cultures had reached confluence. After the third passage, morphologically homogeneous fibroblast-like cells were obtained. These synovial fibroblast cell lines (Passage 4 to 7) were incubated at a density of 1 × 10^5 ^cells/ml in 10% FBS/DMEM and after 24 h, in 1% FBS/DMEM overnight.

### Preparation of nuclear and cytoplasmic extracts

Nuclear and cytoplasmic extracts were obtained from total synovial tissue specimens (333 mg) of patients and cultured RASFs (3.2 × 10^6 ^cells), using the Nuclear Extract kit (Active Motif, Carlsbad, CA, USA) according to the manufacturer's instructions. RASFs were incubated with or without 10 ng/ml of recombinant TNFα (R & D Systems, Minneapolis, MN, USA) at the indicated time (0, 3, 6, 12, 24 h) and (0, 24, 48 h) before extraction. Supernatants were harvested as cytoplasmic fractions. Pellets were resuspended in 100 μl (synovial tissue) and 25 μl (RASFs) of Complete Lysis Buffer and centrifuged at 14,000 × *g *for 10 minutes at 4°C; supernatants were saved as the nuclear fractions. The protein concentration of each sample was measured (Bradford Bio-Rad Protein Assay kit, Bio Rad, San Diego, CA, USA), with bovine serum albumin used as a standard.

### Measurement of HDAC activity

HDAC activity was measured with a non-isotopic assay that used a fluorescent derivative of epsilon-acetyl lysine (HDAC Fluorescent Activity Assay Kit, Biomol, Plymouth Meeting, PA, USA), according to the manufacturer's instructions. Briefly, 6 μg nuclear protein was diluted in assay buffer and incubated at 37°C with cell extracts and trichostatin A (TSA), a classic HDAC inhibitor, and then the HDAC reaction was initiated by the addition of Fluor-de-Lys substrate. After 10 minutes, Fluor-de-Lys Developer was added, and the mixture was incubated for another 10 minutes at room temperature. Fluorescence was measured using a microplate reader (DS Pharma Biomedical, Suita, Osaka, Japan) with excitation at 380 nm and emission at 460 nm. The HDAC activity was expressed as arbitrary fluorescence units. The results are expressed as micromolar values of the provided standard per 6 μg of protein.

### Measurement of HAT activity

HAT activity was measured using a commercially available, non-radioactive HAT activity assay kit (Active Motif, Co., Ltd, Carlsbad, CA, USA), according to the detailed instructions provided by the manufacturer. Nuclear extracts were prepared from synovial tissues obtained from five OA and five RA patients. Briefly, 3 μg nuclear protein was incubated in HAT assay buffer containing 0.5 mM acetyl-CoA and histone H3 peptide (5.16 μg) for 30 minutes at room temperature. The reaction was stopped by adding the stop solution, and after adding the complete developing solution the mixture was incubated for another 15 minutes at room temperature. Fluorescence was measured using a microplate reader (DS Pharma Biomedical) with excitation at 380 nm and emission at 460 nm. The HAT activity was expressed as arbitrary fluorescence units. The results are expressed as micromolar values of the provided standard per 3 μg of protein.

### Immunoassays for TNFα

TNFα plays a central role in regulating pro-inflammatory and anti-inflammatory cytokines in normal, OA and RA synovial tissue. The concentration of TNFα in the cytoplasmic fraction of total synovial tissue was measured by the quantitative sandwich enzyme-linked immunosorbent assay (ELISA) using cytokine-specific capture and biotinylated detection mAbs and recombinant cytokine proteins (all from BD Biosciences, San Jose, CA, USA) according to the manufacturer's protocol. The detection limit of the kit for TNFα was 7.8 pg/ml.

### Quantitative real-time RT-PCR for HDAC1-8 and TNFα

Total RNA was extracted from total synovial tissue 30 mg in size with the use of an RNeasy Fibrous Tissue Mini Kit (Quiagen, Valencia, CA, USA) and was extracted from RASFs (n = 3) after stimulation by recombinant TNFα (10 ng/ml) at the indicated time (0, 3, 6, 24 h) using TRIzol according to the instructions of the manufacturer (Invitrogen, Carlsbad, CA, USA). Two micrograms of total RNA was reverse transcribed to complementary DNA (cDNA) with random primers according to the manufacturer's protocol (Toyobo, Osaka, Japan).

Quantitative real-time RT-PCR analysis was performed using a LightCycler Rapid Thermal Cycling system (Roche Diagnostics, Indianapolis, IN, USA), according to a previously reported protocol [25-27]. The PCR mixture consisted of 1 × SYBR Green PCR Master Mix, which includes DNA polymerase, SYBR Green I dye, dNTPs (including dUTP), PCR buffer, 10 pmoles of forward and reverse primers, and cDNA of samples, in a total volume of 20 μl. Amplification of a housekeeping gene, β-actin, was used for normalizing the efficiency of cDNA synthesis and the amount of RNA applied. The sequences of the oligonucleotide primers (HDAC1-8) used are shown in Table 2 and TNFα primer (Roche Diagnostics) was used.

**Table 2:** Primer pairs for PCR detection

HDAC1	F	AGACAGCTGTGGCCCTGGATAC
	R	CGGCAGCATTCTAAGGTTCTCAA
HDAC2	F	TGCAGTTGCCCTTGATTGTGA
	R	ATCTGGACACCAGGTGCATGAG
HDAC3	F	AGAGTGGCCGCTACTACTGTCTGAA
	R	TGGGTTGGTAGAAGTCCACTACCTG
HDAC4	F	GAGCAGGAGCTGGAGAAG
	R	AATGCAGTGGTTCAGATTCC
HDAC5	F	GTGTCTCGGCTCTGCTCAGTGTAG
	R	TTTGCTGCAAGACTGCCTCATC
HDAC6	F	GTCTACTGTGGTCGTTACATC
	R	GGCCTGACAGTAGTAACAC
HDAC7	F	CGGGTGCACAGTAAATAC
	R	CCAGAGCCTTAGAGATTCATA
HDAC8	F	TGACGGAATGTGCAAAGTAGCAA
	R	TCAAATTTCCGTCGCAATCGTA
β-actin	F	TTCCTGGGCATGGAGTCCT
	R	AGGAGGAGCAATGATCTTGATC

### Western blotting for class I HDACs

Nuclear extracts (20 μg of total protein/lane from synovial tissue and 5 μg of total protein/lane from RASF) were subjected to SDS-PAGE using a 5 to 12% gradient gel, and then transferred onto nitrocellulose membranes (Advantech Co., Ltd, Tokyo, Japan). The membranes were blocked with 5% skim milk and 0.05% Tween 20 in TBS (pH 7.4) for 1 h. and were then incubated with primary antibody in 0.05% Tween 20 in TBS (pH 7.4) overnight at 4°C with polyclonal antibodies against HDAC1, 2, 3, 8 (Santa Cruz Biotechnology, Santa Cruz, CA, USA, HDAC1:SC-7872, HDAC2:SC-7899, HDAC3:SC-11417, HDAC8:SC-11405) and lamin A (Abcam Ltd., Cambridge, UK, ab8980). After washing, blots were stained with appropriate horseradish peroxidase-conjugated secondary antibody. Immunoreactive bands were detected by enhanced chemiluminescence (ECL plus solution, Amersham Pharmacia Biotech, Piscataway, NJ, USA). Protein expression level was quantitated by densitometric analysis as previously reported [28]. Briefly, the intensity of the bands were measured by Image J software, Bethesda, MD, USA [29] and lamin A, a nuclear protein, was used for loading controls.

### Statistical analysis

All results are expressed as means ± SE. Statistical significance of differences between two groups was determined by the Mann-Whitney U test. Other analysis was performed to compare differences among groups by one-way analysis of variance followed by the Bonnferoni correction. A *P*-value of less than 0.05 was considered statistically significant.

## Results

### Measurement of HDAC activity in RA, OA and normal synovial tissues

**Figure 1:**
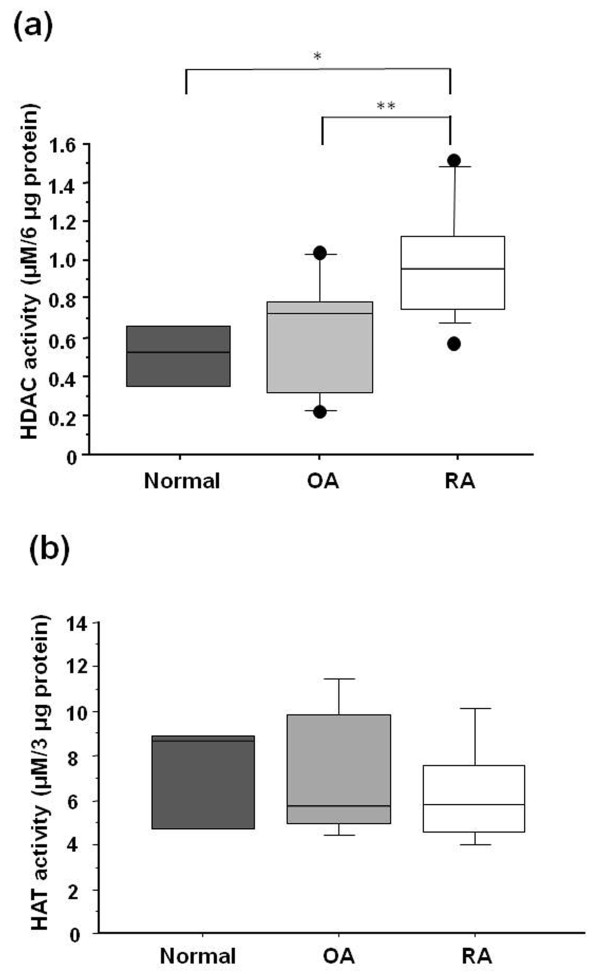
**Total nuclear histone deacetylase (HDAC) and histone deacetylase (HAT) activity in synovial tissues**. **(a) **Nuclear HDAC activity in synovial tissues that were obtained from rheumatoid arthritis (RA, n = 14) patients was significantly increased compared to that from normal controls (n = 3) and osteoarthritis (OA, n = 12). In the box (interquartile range, IQR) and whisker (maximum and minimum) plots, the horizontal line inside the box denoted median and the filled circles denote outliers outside IQR ± 1.5 × IQR. * = *P *< 0.05 versus normal controls, ** = *P *< 0.05 versus OA. **(b) **Nuclear HAT activity was measured in synovial tissues that were obtained from normal controls (n = 3), OA (n = 5) and RA patients (n = 5). There was no significant difference between normal controls, OA and RA synovial tissues.

Total nuclear HDAC activity (expressed as micromolar values of the deacetylated HDAC substrate standard per 6 μg of protein) in samples of synovial tissue from RA patients (n = 14) was 0.96 ± 0.08 μM of the HDAC standard. This level of activity was significantly higher than those from OA (n = 12, HDAC activity; 0.62 ± 0.09, *P *= 0.0052) and from normal controls (n = 3, HDAC activity; 0.50 ± 0.04, *P *= 0.015) (Figure 1a).

### Measurement of HAT activity in RA, OA and normal synovial tissues

Total nuclear HAT activity was measured in synovial tissues from normal controls (7.04 ± 1.78 μM, n = 3), from OA (7.23 ± 1.35 μM, n = 5) and from RA (6.28 ± 1.06 μM, n = 5) (Figure 1b). There was no significant difference between normal controls, OA and RA synovial tissues (Figure 1b). We examined the ratio of HDAC to HAT activity on the same patients (OA: n = 5, RA: n = 5), but failed to show the significant difference in the ratio of HDAC to HAT activity between OA and RA groups. This might be partly due to small data set, but, at least, HDAC/HAT was not shifted toward histone hyperacetylation.

### Relationship between nuclear HDAC activity and cytoplasmic TNFα levels

**Figure 2:**
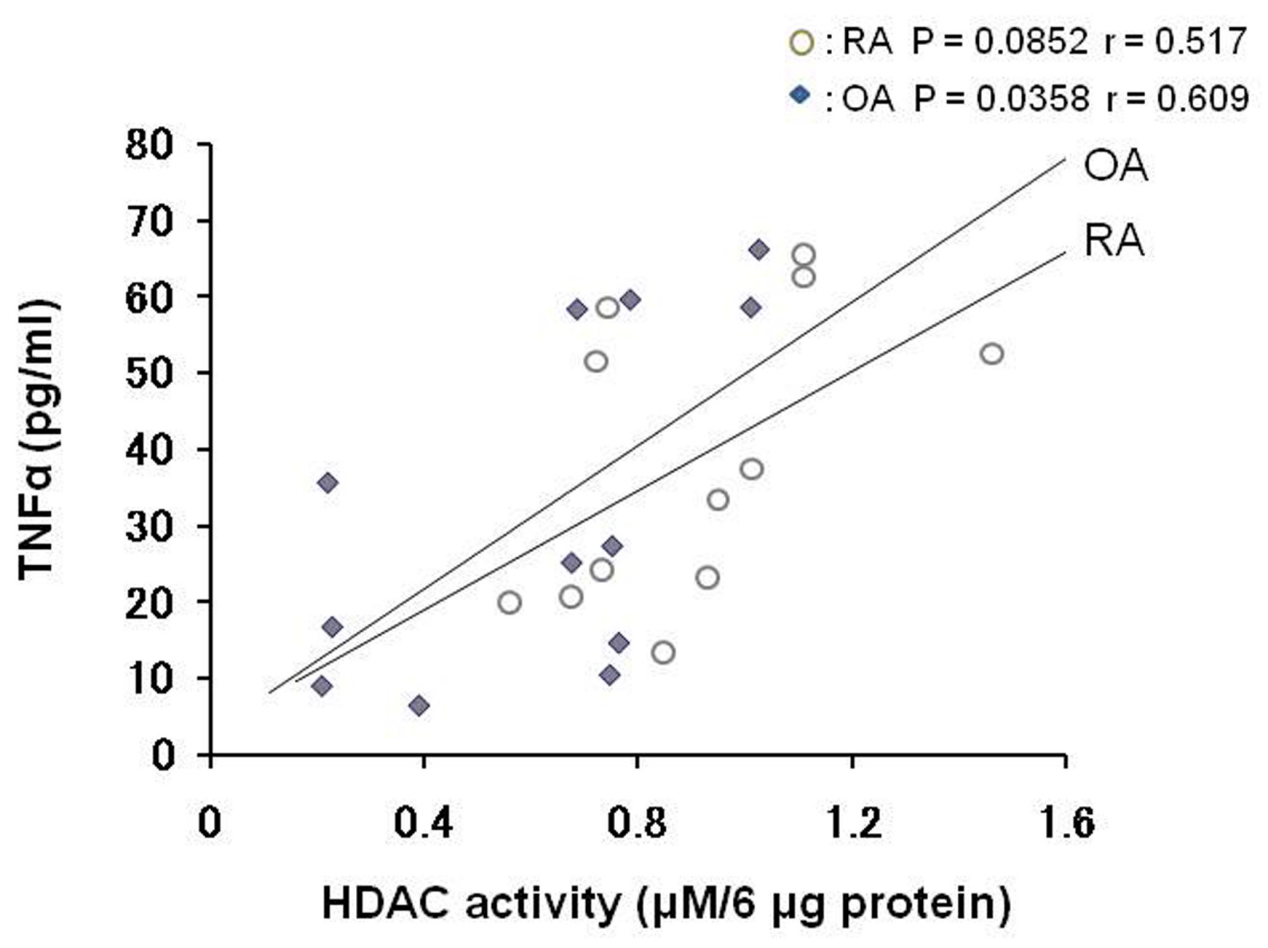
**Correlation between the amount of cytoplasmic TNFα and nuclear HDAC activity in synovial tissues**. Both cytoplasimc TNFα and nuclear HDAC activity were measured in synovial tissues that were obtained from osteoarthritis (n = 12), and rheumatoid arthritis (n = 12) patients. The cytoplasmic fraction was obtained during the isolation of the nuclear fraction, and analyzed by ELISA.

To establish the relationship between nuclear HDAC activity and cytoplasmic TNFα levels directly, we measured TNFα of the cytoplasmic fraction which was obtained by preparing nuclear extracts of OA (n = 12) and RA synovial tissues (n = 12). The amount of cytoplasmic TNFα tended to correlate with nuclear HDAC activity (OA: correlation coefficient r = 0.609, *P *= 0.0358, RA: r = 0.517, *P *= 0.0852) (Figure 2).

### Class I, class II HDACs and TNFα mRNA expressions in total synovial tissue

**Figure 3:**
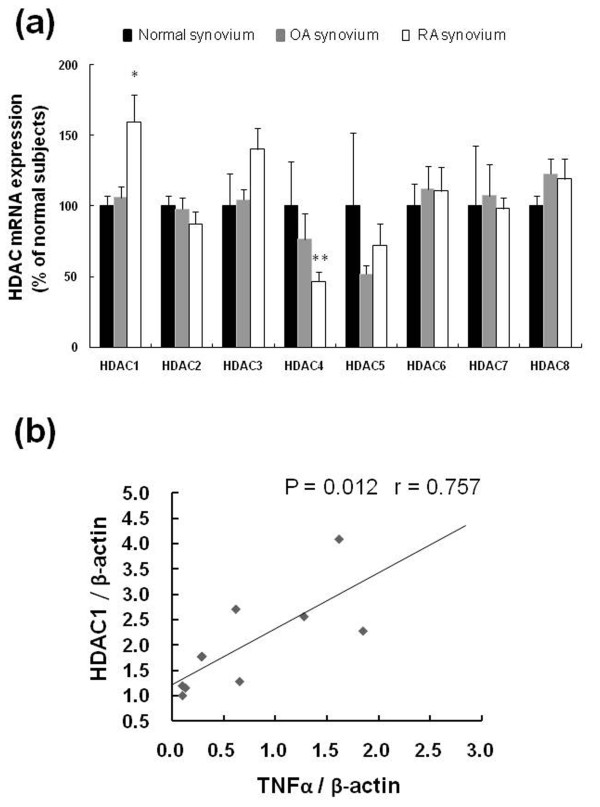
**HDAC1-8 mRNA expression and correlation between TNFα mRNA and HDAC1 mRNA expressions in synovial tissues**. Total RNA was extracted from total synovial tissues that were obtained from normal controls (n = 3), osteoarthritis (OA, n = 8) and rheumatoid arthritis (RA, n = 9) patients. **(a) **HDAC1-8 mRNA expression was analyzed by quantitative real-time PCR analysis as described in *Materials and Methods*. * = *P *< 0.05 versus OA, ** = *P *< 0.05 versus normal controls. **(b) **Correlation between TNFα mRNA expression and HDAC1 mRNA in RA synovial tissues (n = 10).

To investigate the expression profiles of class I and class II HDACs in RA (n = 9), OA (n = 8) and normal controls (n = 3) synovial tissues, mRNA levels of HDAC1 to 8 were evaluated by quantitative real-time PCR. RA synovial tissues expressed high levels of HDAC1 compared to OA (*P *= 0.018), and normal controls (Figure 3a). HDAC4 mRNA levels were significantly higher in normal controls than in RA (*P *= 0.016). TNFα mRNA expression was measured in RA synovial tissue. The data showed significant positive correlation between TNFα and HDAC1 mRNA in RA synovial tissue (n = 10) (correlation coefficient r = 0.757, *P *= 0.012) (Figure 3b).

### Nuclear expression of class I HDACs in synovial tissue

**Figure 4:**
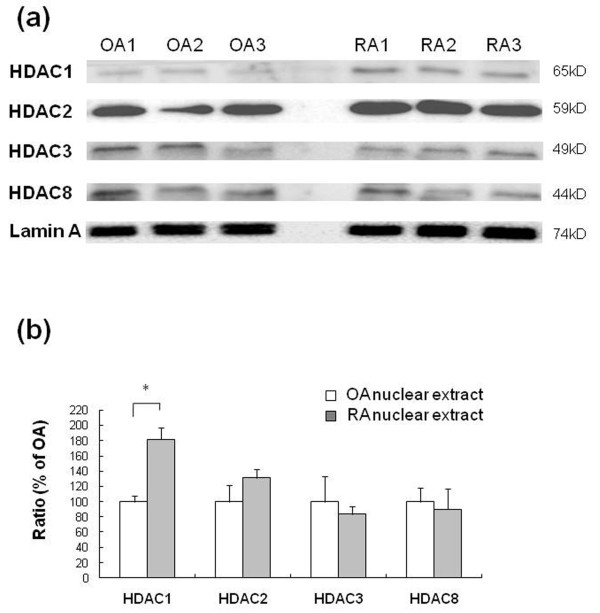
**Results of Western blot analysis for nuclear class I HDACs protein expressions in synovial tissues**. (**a**) Class I HDACs (HDAC1, 2, 3, 8) protein expressions in synovial tissues that were obtained from osteoarthritis (OA, n = 3) and rheumatoid arthritis (RA, n = 3) synovial tissues. Lamin A, nuclear membrane protein, served as loading control. (**b**) Densitometric analysis class I HDACs protein in OA and RA synovial tissue was The data were plotted as means ± SE. * = *P *< 0.05 versus OA.

We performed Western blotting for nuclear class I HDACs in synovial tissue. The level of nuclear HDAC1 protein expression was higher in RA synovial tissue compared with OA synovial tissue (Figure 4a). Western blots were quantified by Image J software [29]. The level of expression of HDAC1 by normalizing to the band density of nuclear membrane protein lamin A was significantly higher in RA than OA synovial tissue (*P *= 0.0495) (Figure 4b) (see Additional file 1).

### Measurement of HDAC activity, class I HDACs mRNA expressions and nuclear expressions in RASFs after treatment with TNFα

**Figure 5:**
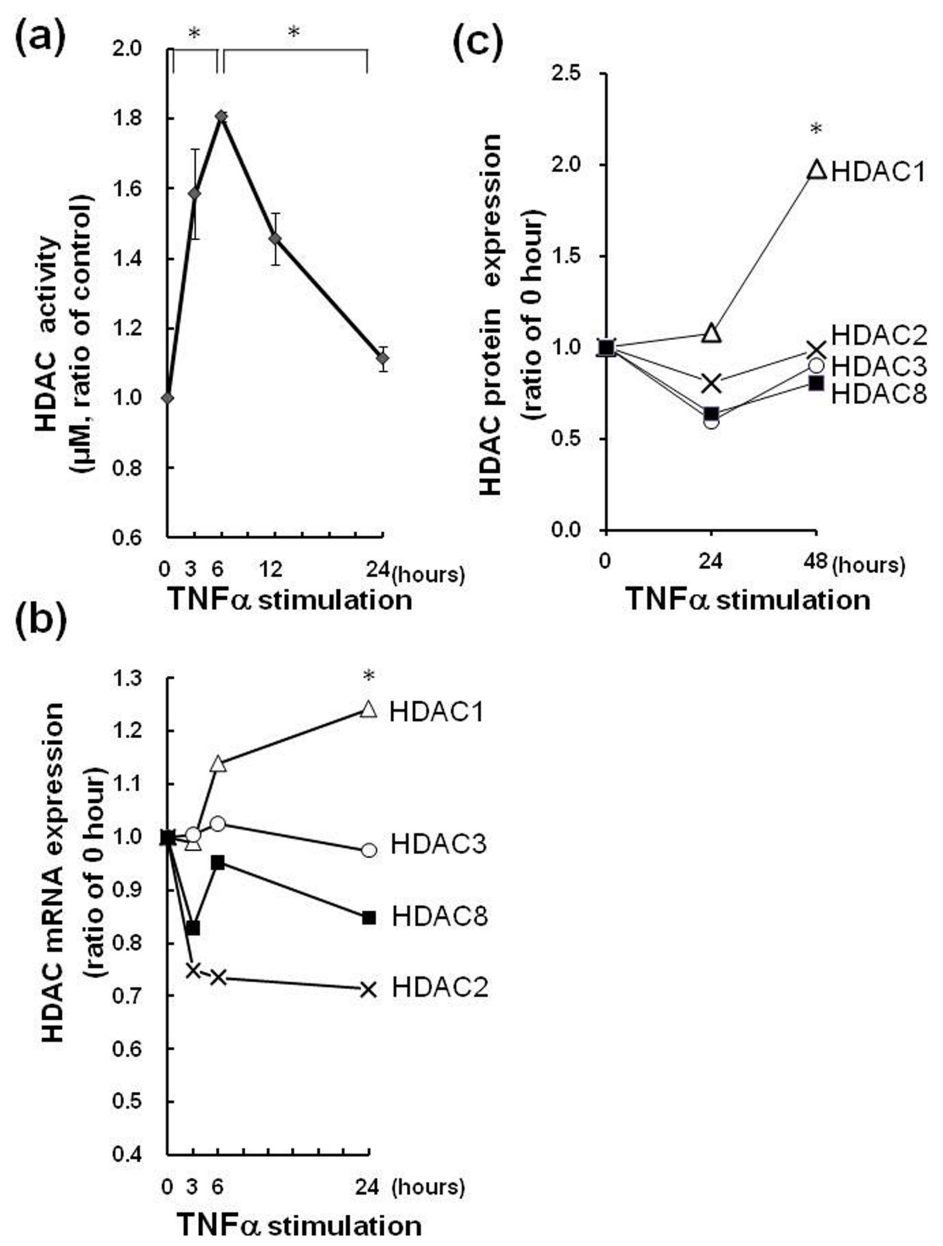
**Nuclear HDAC activity, class I HDACs mRNA and protein expressions in RASFs after TNFα treatment**. RASFs (n = 3) were treated with TNFα (10 ng/ml) and total nuclear protein was extracted at the indicated time points. **(a) **Nuclear HDAC activity was elevated significantly at 6 h. The data were plotted as means ± SE. * = *P *< 0.001 versus time 0 h, 24 h. **(b) **The expression of HDAC1 in RASFs was increased after TNFα treatment, while the expressions of other class I HDACs were not elevated. The increase of mRNA in HDAC1 at 24 h was significantly greater than that in other class I HDACs. For real-time PCR analysis, levels of mRNA were normalized against actin expression and compared with 0 h. * = *P *< 0.05 versus HDAC2, 3, 8. **(c) **Quantitative results of Western blot analysis of nuclear class I HDACs protein expression in RASFs. The band intensity of class I HDACs was measured by Image J software. For analysis, levels of protein expression were normalized by lamin A and compared with 0 h. * = *P *< 0.05 versus HDAC3 and HDAC8 at 48 h.

Treatment by TNFα significantly increased nuclear HDAC activity in RASFs (n = 3) and peaked at 6 h (*P *= 0.0001 vs 0 and 24 h) (Figure 5a), indicating that TNFα stimulation seems to be associated with nuclear HDAC activity in RASFs. Next, the change of mRNA expression in the class I HDACs after TNFα stimulation was analyzed. The expression of HDAC1 in RASFs (n = 3) was increased after TNFα treatment; while the expressions of other class I HDACs were not elevated through the time course (0, 3, 6, 24 h) (Figure 5b). When the relative mRNA expressions at 24 h after stimulation were compared among class I HDACs, the increase of mRNA in HDAC1 was significantly greater than that in other class I HDACs (*P *= 0.02). We performed Western blotting for nuclear class I HDACs in RASFs. Western blots were quantified by Image J software. The nuclear HDAC1 protein expression in RASFs (n = 3) was elevated compared to other class I HDACs (HDAC2, HDAC3, HDAC8) after TNFα treatment though the time course (0, 24, 48 h). The level of protein expressions by normalizing to the band density of nuclear membrane protein lamin A at 48 h after TNFα treatment tended to increase in HDAC1 (*P *= 0.0654; versus HDAC2, *P *= 0.049; versus HDAC3, *P *= 0.0354; versus HDAC8) (Figure 5c) (see Additional file 2).

## Discussion

Previous reports indicated that HDAC inhibitors exhibit anti-inflammatory properties, and might play a beneficial role in the treatment of inflammatory diseases, such as ulcerative colitis, lupus erythematosus and hepatic injury [14,30-32]. In contrast, HDAC inhibitors have been shown to enhance lung and microglial inflammation, suggesting that HDAC inhibitors might modulate inflammation in a cell type-specific manner. We demonstrated recently that FK228, a specific class I HDAC inhibitor, prevents the *in vivo *proliferation of RASFs and ameliorates the pathological changes of autoantibody-mediated arthritis in mice [19]. These results strongly suggested that modulation of the transcriptional activity of specific promoters in response to the local release or perturbation of chromatin structure, by treatment with HDAC inhibitors, could effectively prevent the synovial proliferation and joint destruction seen in human RA. It is still not known however, which HDAC was a candidate gene that should be targeted in the process of human RA inflammation.

In this study, we demonstrated that total nuclear HDAC activity is increased in samples of human RA synovial tissues compared to that in samples of OA and normal subjects. Interestingly, our results were the opposite of that reported by Huber *et al *[12,33]. The following factors may lead to the discrepancies between the two studies. First, they obtained the synovial samples during joint replacement surgery of seven RA patients, six OA patients and three control subjects. In RA, three patients received TNFα blockade and all normal samples were obtained via autopsies. As synovial tissue under TNFα blockade would not represent the regular RA inflammation, and HDAC/HAT activity might change after mortality, we excluded the patients receiving TNFα blockade treatment, and all samples were obtained at surgery. Second, they demonstrated lower levels of HDAC1, and HDAC2 protein in RA synovium than in OA by Western blotting of whole cell lysates, with α-tubulin as an internal control. Because HDAC1 and HDAC2 are localized mainly at the cell nuclei, we compared the nuclear protein levels of HDACs between RA and OA, with lamin A as an internal control, and showed a significant increase of HDAC1 protein in RA cells. This discrepancy might have resulted partly from the difference in the number of samples.

In chronic inflammation diseases, such as RA, TNFα is a master cytokine that governs the disease process by inducing a variety of inflammatory mediators through activation of the transcription factor, NF-κB, and the MAP kinase cascade. We examined the relationship between nuclear HDAC activity and cytoplasmic TNFα in synovial tissue (Figure 2). They were significantly correlated in OA synovial tissue, whereas they did not reach statistically significant correlation in RA synovial tissues (r = 0.517, *P *= 0.0852). These data imply a limitation of the current study that nuclear HDAC activity and cytoplasmic TNFa levels in synovial tissues from RA patients can be affected by medical treatments with DMARDs or corticosteroid.

The previous study reported that TNFα modestly activated HDAC activity in airway smooth muscle cells [34]. Our *in vitro *study indicated that stimulation by TNFα up-regulated HDAC activity in RASFs, suggesting the downstream role of HDAC in exacerbation of the inflammation, and that the inhibition of HDAC activity results in the suppression of arthritis. Therefore, blockage of TNF by biologic agents might result in the inhibition of HDAC activation in synovial tissue.

On the other hand, anti-inflammatory effects shown by inhibition of HDAC activity [15,19] might be associated with the inhibition of the TNFα-induced NF-κB pathway. In non-small cell lung cancer (NSCLC), the HDAC inhibitor superoylanilide hydroxamic acid (SAHA) displayed antitumor efficacy by delayed IκB-α phosphorylation [35]. Butyrate, a classical HDAC inhibitor, inhibited NF-κB DNA binding within 30 minutes of TNFα stimulation, consistent with the inhibition of NF-κB nuclear translocation in colonocytes [36]. The influence of HDAC inhibitors on transcriptional co-factors or/and co-activators after DNA binding of NF-κB still requires further investigation in RA.

Next, we attempted to investigate HDAC specificity in RA inflammation. In RA synovial tissues, we demonstrated that HDAC1 was specifically up-regulated in mRNA expression and protein levels. Western blot analysis of class I HDACs in synovial tissues showed that the expression of HDAC1 protein was significantly increased in RA lesions, compared with OA lesions. In RASFs, only HDAC1 mRNA and HDAC1 protein expression among class I HDACs increased through the time courses after TNFα stimulation, suggesting that HDAC1 overexpression might be associated with the enhanced inflammatory reaction. A previous report showed the effects of therapeutic administration of the HDAC inhibitor, SAHA and MS-275 on disease progression and joint destruction in collagen-induced arthritis in rat and mouse models [22]. Although SAHA exhibited moderate prophylactic efficacy, but could not inhibit the onset of arthritis, MS-275 displayed dramatic anti-rheumatic activities. In prophylactic intervention, high doses of MS-275 prevented bone erosion, and displayed dramatic anti-rheumatic activities. The authors concluded that the superior anti-inflammatory effects of MS-275 might be due to its specificity towards class I HDACs, especially HDAC1.

The disruption of both HDAC1 alleles results in embryonic lethality, as a result of severe proliferation defects and retardation in development [37]. Published data indicate that HDAC1 knockdown by siRNA induces a mitotic defect, cell growth inhibition, and an increased percentage of apoptotic cells in human tumor cells [38]. These findings indicate that HDAC1 has important roles in development and proliferative disease, which may include tumor-like proliferative inflammatory disease, such as RA. HDAC1 target genes include *Bax*, cytokeratin 18, p21^WAF1/Cip1^, p27^KIP1^, p16^INK4a ^and p53 [18,37,39-41]. Especially, several studies suggest that the tumor suppressor gene p53 is a key regulator in rheumatoid inflammation. p53 mutations in RA synovial tissue and RASF have been reported, although there is some variability in the number of mutations identified [42,43]. Loss of p53 function in RASF and in collagen antibody-induced mice enhances proliferation, cartilage invasion and anchorage-independent growth while suppressing apoptosis, thereby recapitulating the rheumatoid phenotype [38,44]. It is known that HDAC1 deacetylates p53 *in vitro *and *in vivo*, and down-regulates p53 transcriptional activity [45,46]. Effective degradation of p53 is mediated by the ubiquitin ligase Mdm2, as well as in RA [47], and Mdm2 can promote p53 deacetylation by recruiting a complex containing HDAC1 [48]. Most recently, Horiuchi *et al. *also showed HDAC1 is overexpressed in RASF compared to OA synovial fibroblasts [41]. Knockdown of HDAC1 and HDAC2 by siRNA resulted in increased expression of p16, p21, and p53, and decreased cell counts and cell proliferation, and increased apoptosis in RASF. These data cumulatively support the idea that HDAC1 might be involved in RA pathogenesis by regulating the cell cycle of synovial tissue, and might contribute synovial inflammation.

## Conclusions

The relationship between histone acetylation and RA pathogenesis has not been elucidated. Our results indicate that higher HDAC activity might be linked with higher amounts of cytoplasmic TNFα in RA synovial tissues. Among HDACs, increased activity and expression of nuclear HDAC1 in synovial cells might play a role in RA inflammation.

## Abbreviations

DMARDs: disease-modifying anti-rheumatic drugs; DMEM: Dulbecco's modified Eagle's medium; FBS: fetal bovine serum; HAT: histone acetyl transferase; HDAC: histone deacetylase; IL: interleukin; MTX: methotrexate; NF: nuclear factor; NSAIDs: nonsteroidal anti-inflammatory drugs; NSCLC: non-small cell lung cancer; OA: osteoarthritis; RA: rheumatoid arthritis; RASF: synovial fibroblasts from rheumatoid arthritis; SAHA: superoylanilide hydroxamic acid; TNF: tumor necrosis factor; TSA: Trichostatin A.

## Competing interests

The authors declare that they have no competing interests.

## Authors' contributions

TK was responsible for the experiments and data analysis and wrote the report. KN was responsible for the planning of the research and wrote the manuscript. KT and HO participated in the design of the study. KS, YK, and JI assisted with the experiments. SH, YN and HM contributed to the planning of the research. All authors read and approved the final manuscript.

## Supplementary Material

Additional file 1 **Results of Western blot analysis for nuclear class I HDACs protein expressions in synovial tissues**. Nuclear class I HDACs (HDAC1, 2, 3, 8) protein expressions were obtained from synovial tissues of RA (n = 1) and OA (n = 1) [29]. Click here for file

Additional file 2 **Results of Western blot analysis for nuclear class I HDACs protein expression in RASFs after TNFα treatment**. Nuclear class I HDACs (HDAC1, 2, 3, 8) protein expressions that were obtained from RASFs (n = 3). RASFs were treated with TNFα (10 ng/ml) at the indicated time points. Click here for file
