# Supplementary figures and images for: Increased activity and expression of histone deacetylase 1 in relation to tumor necrosis factor-alpha in synovial tissue of rheumatoid arthritis
Source: Arthritis Res Ther. 2010 Jul 7;12(4):R133. doi: 10.1186/ar3071 (PMC2945023; doi:10.1186/ar3071)

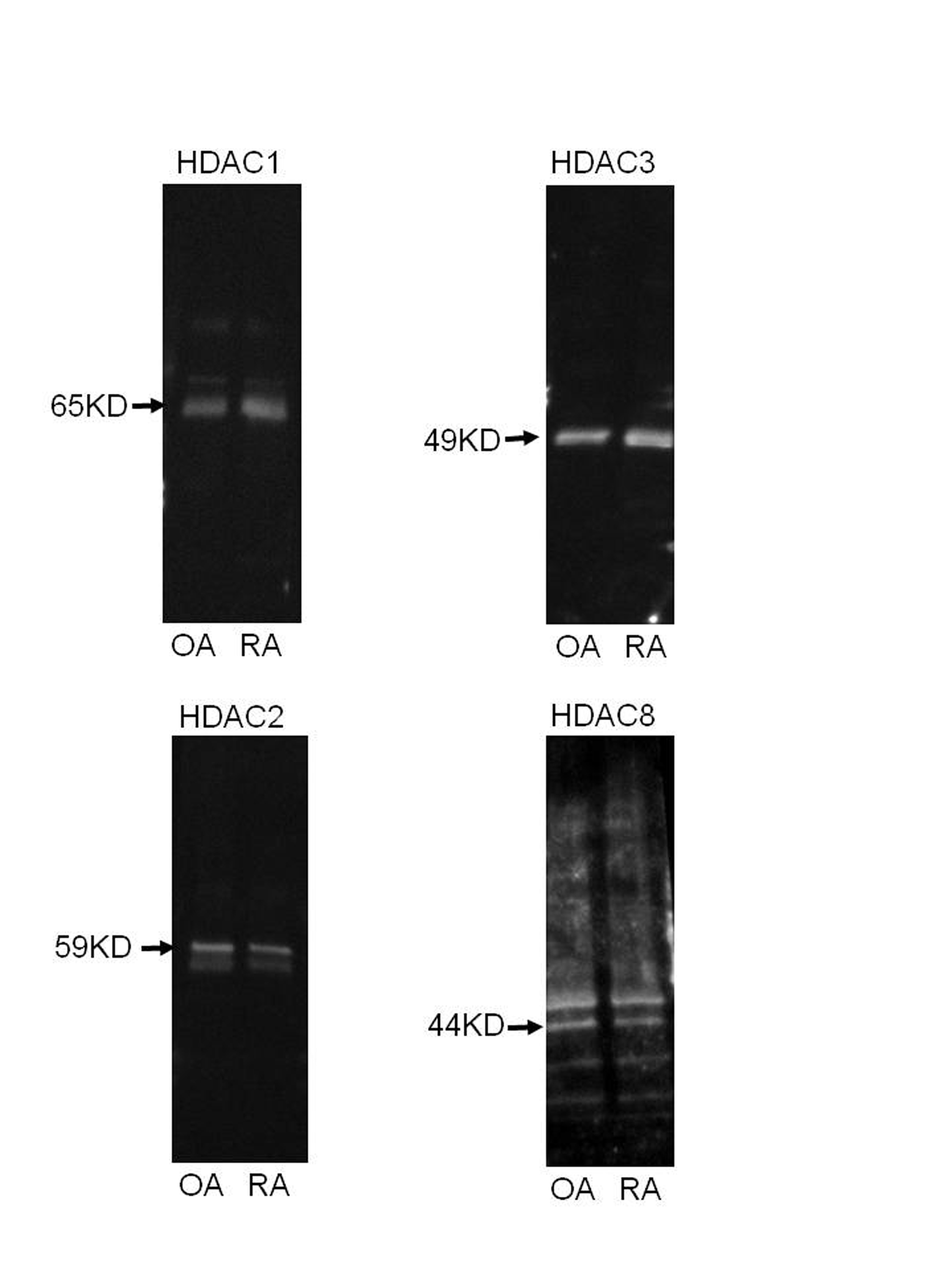

Supplement: Additional file 1 — Results of Western blot analysis for nuclear class I HDACs protein expressions in synovial tissues. Nuclear class I HDACs (HDAC1, 2, 3, 8) protein expressions were obtained from synovial tissues of RA (n = 1) and OA (n = 1) [29]. [file ar3071-S1.JPEG]

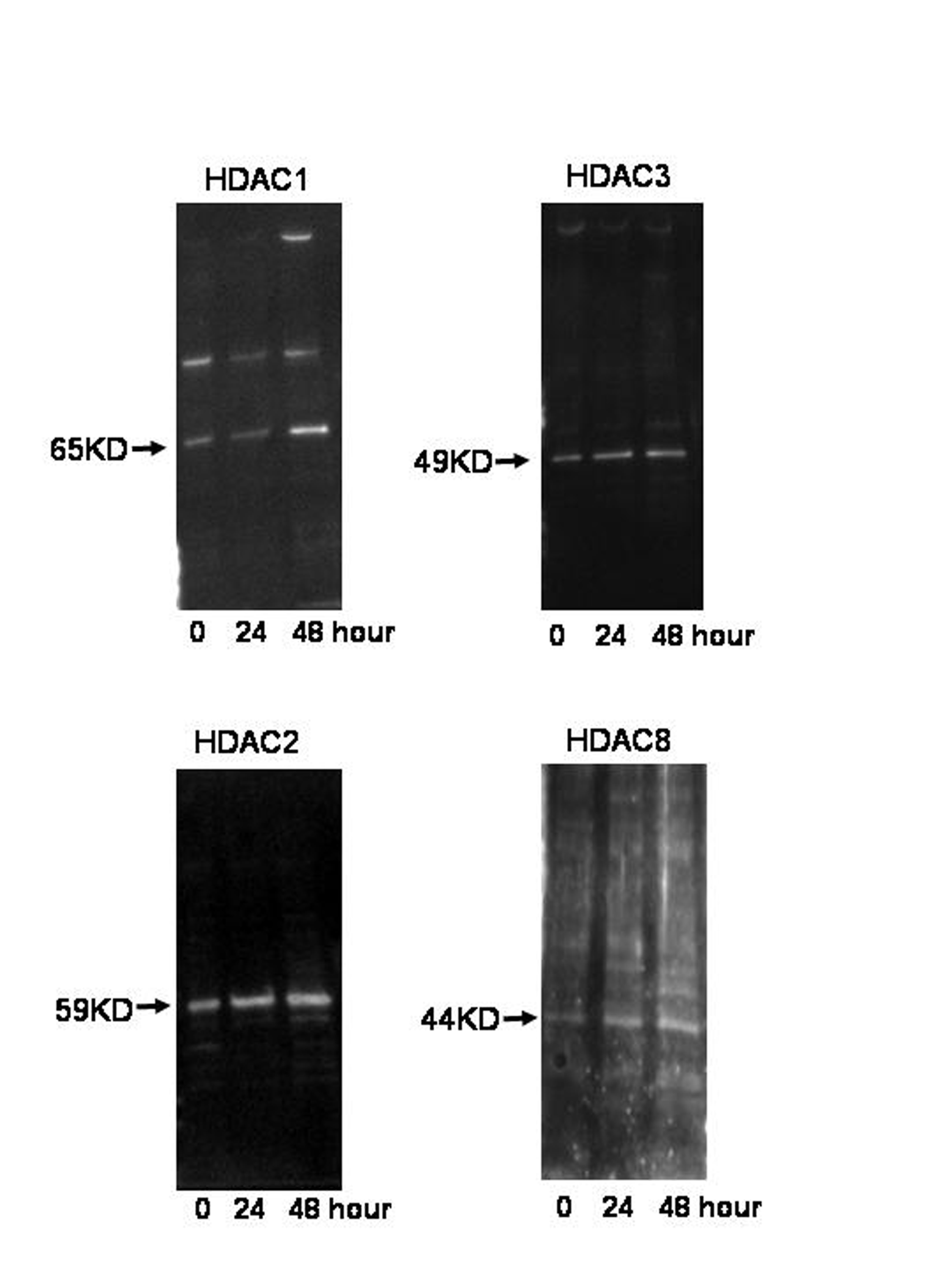

Supplement: Additional file 2 — Results of Western blot analysis for nuclear class I HDACs protein expression in RASFs after TNFα treatment. Nuclear class I HDACs (HDAC1, 2, 3, 8) protein expressions that were obtained from RASFs (n = 3). RASFs were treated with TNFα (10 ng/ml) at the indicated time points. [file ar3071-S2.JPEG]
